# The mediating advantage of social capital: examining the pathways from physical exercise to subjective well-being

**DOI:** 10.3389/fpsyg.2026.1749855

**Published:** 2026-01-22

**Authors:** Zhoujie Mao

**Affiliations:** Department of Academic Affairs, Zhejiang Police College, Hanghzou, Zhejiang, China

**Keywords:** health capital, mediating mechanisms, physical exercise, social capital, well-being

## Abstract

**Introduction:**

Subjective well-being is a crucial indicator of individual quality of life and mental health. Physical exercise is widely recognized as a beneficial behavior for enhancing well-being, yet the underlying mechanisms—particularly the relative importance of social capital versus health capital—remain to be fully elucidated. The aim of this study was to investigate the impact of physical exercise on subjective well-being and to compare the dual mediating roles of social capital and health capital using nationally representative panel data.

**Methods:**

This study utilized three waves of panel data from the China Family Panel Studies (CFPS) spanning 2018 to 2022 and applied mixed regression models, fixed effects models, and Bootstrap mediation analysis to systematically examine the impact of physical exercise on subjective well-being and its underlying mechanisms.

**Results:**

The results show that: (1) A robust positive association between physical exercise and well-being was identified. However, after controlling for time-invariant individual traits in the fixed effects model, the coefficient substantially decreased, suggesting that part of the observed association may reflect stable psychosocial differences across individuals. (2) Mediation analysis revealed that the social capital pathway exerted the strongest mediating effect (accounting for 44.6% of the total effect), significantly exceeding that of the health capital pathway (14.1%). This indicates that physical exercise primarily influences well-being through mechanisms of social integration. (3) The depression risk pathway also exhibited a significant mediating effect (38.7%), highlighting the beneficial role of physical exercise in psychological and emotional well-being.

**Conclusion:**

These findings imply that public health policies should emphasize the social embedding of physical exercise by promoting community-based and group activities to more effectively achieve the dual goals of health promotion and enhancement of subjective well-being. Additionally, this empirical study set in the context of Chinese collectivist culture provides critical evidence for cross-cultural comparisons of sociocultural factors and health behavior mechanisms, thereby expanding the culturally specific perspective in well-being research.

## 1 Introduction

Subjective well-being, a core indicator of individual quality of life, not only reflects mental health status but is also closely linked to overall societal welfare. In recent years, against the backdrop of an accelerating pace of life and increasingly prominent mental health issues, enhancing well-being has become a significant topic in both public health and psychological research. Physical exercise, as a low-cost and easily implementable lifestyle intervention, is widely recognized to be positively associated with well-being. Although numerous studies have explored the relationship between physical exercise and well-being, several research gaps and unresolved questions remain.

Physical exercise is not merely an isolated physical activity but a social practice. The social spheres it creates, the accumulated social relationships, and the health capital generated through this process, and how these factors collectively influence individual well-being, constitute a critical area for further inquiry. Although existing studies have confirmed the mediating roles of social capital and health capital in the relationship between physical exercise and well-being (Huang et al., 2024; Beauchamp et al., 2018; Wang et al., 2021), several limitations remain. First, most research tends to focus on a single physiological or psychological pathway, lacking comprehensive comparison and integration of multiple mediating pathways—especially the social capital pathway. Second, theoretical explanations regarding how physical exercise influences well-being through social capital remain underdeveloped, often limited to descriptive associations. Third, there is a scarcity of systematic investigations into how cultural contexts might moderate the mechanisms linking physical exercise and well-being, resulting in insufficient cross-cultural theoretical dialogue.

## 2 Literature review and research hypotheses

### 2.1 Definition and measurement of core concepts

Social capital refers to the trust, support, reciprocal norms, and collective action capabilities that individuals can access through their social relationship networks. Based on the CFPS questionnaire, social capital is operationalized across three dimensions: social interaction, social status, and social trust. Health capital denotes an individual’s subjective evaluation and objective accumulation of their physical and mental health status (Steptoe et al., 2015). In this study, self-rated health is adopted as the primary measure of health capital. Although self-rated health may be influenced by subjective judgment, it is widely accepted by scholars as a reliable assessment tool and frequently used in relevant research (Xia et al., 2024; Wang et al., 2022). Depression risk is defined as a psychological vulnerability characterized by persistent low mood, loss of interest, and a range of associated cognitive and somatic symptoms (Kessler and Bromet, 2013), serving as a key negative predictor of subjective well-being. The CFPS employs the 8-item CES-D scale to measure depression risk.

### 2.2 The impact of physical exercise on well-being

The beneficial effects of physical exercise on well-being are multifaceted and operate through multiple pathways. Empirical evidence indicates that consistent exercise not only improves physical health but also alleviates psychological stress and elevates life satisfaction. From a physiological perspective, the neurochemical hypothesis posits that exercise stimulates the release of endorphins, dopamine, norepinephrine, and serotonin in the brain. The increased levels of these neurotransmitters directly elicit positive affect, reduce anxiety and depressive symptoms (i.e., decrease negative affect), and contribute to the phenomenon commonly known as the “runner’s high” (Boecker et al., 2008). From a psychological standpoint, activities such as completing a run, lifting heavier weights, or observing improvements in body composition provide immediate achievement feedback. This process significantly enhances self-efficacy—the belief in one’s capability to succeed—which constitutes a core component of environmental mastery and self-acceptance in Ryff’s (2014) psychological well-being model. Basso and Suzuki (2017) further suggest that exercise offers a temporary respite from daily stressors and negative thoughts, serving as an effective emotion regulation strategy. Therefore, research on the mechanisms underlying the relationship between physical exercise and subjective well-being is evolving from a single physiological model toward an integrative biopsychosocial framework. Based on this evidence, we propose the following hypothesis:

Hypothesis 1: Physical exercise directly enhances individuals' subjective well-being and psychological well-being.

### 2.3 The roles of health capital and depression risk in the influence of physical exercise on subjective well-being

Improvement in health and alleviation of depressive symptoms represent two classic pathways through which physical exercise affects subjective well-being, supported by substantial empirical evidence. Among the elderly population in China, Wang et al. (2021) demonstrated based on CGSS data that physical exercise indirectly enhances subjective well-being by improving self-rated health. This suggests that physical exercise not only promotes well-being indirectly through enhancing physiological functioning but also plays a key role by elevating individuals’ subjective evaluations of their physical and mental states. Redwine et al. (2019) found that both Tai Chi and resistance training significantly reduce depressive symptoms in patients with heart failure. Chinese scholars Yang et al. (2021) validated the mediating effect of psychological resilience on the relationship between physical exercise and mental well-being among community-dwelling older adults.

However, research on these two pathways has certain limitations: First, the theoretical perspective is largely individualized, focusing on physiological and psychological changes within the individual, without sufficiently incorporating the moderating effects of sociocultural contexts. Second, in collectivist cultures, the explanatory power of these pathways may be relatively limited. When subjective well-being is more dependent on social recognition, improvements solely in individual health and mood may not suffice to produce significant enhancements in well-being. This underscores the necessity to examine the relative importance of the social pathway. Accordingly, we propose the following hypothesis:

Hypothesis 2: Health capital and depression risk mediate the relationship between physical exercise and subjective well-being; that is, physical exercise indirectly affects well-being by enhancing health capital and reducing depression risk.

### 2.4 The role of social capital in the relationship between physical exercise and well-being

Social capital—including social networks, social trust, and social participation—is regarded as a key mechanism through which physical exercise impacts subjective well-being. Sylvester et al. (2016) found that team sports significantly enhance participants’ well-being by fostering social support and trust. Based on CFPS data, Huang et al. (2024) indicated that social capital mediates the relationship between physical activity and individual welfare, with collective social capital exerting a more pronounced effect on welfare improvements in education and income dimensions. However, existing studies often treat social capital as just one among multiple mechanisms, lacking theoretical justification and empirical testing regarding its relative importance across diverse pathways.

The collectivist cultural context of China offers a unique theoretical setting to examine the predominance of the social capital pathway. In collectivist societies, individual well-being is more deeply embedded within social relationships, and social harmony and a sense of belonging may contribute more to subjective well-being than personal achievements. Therefore, the social integration function of physical exercise may be amplified within the Chinese context, making its social pathway effect stronger than the physiologically centered individual health pathway. This theoretical inference, however, remains inadequately tested using nationally representative longitudinal data. Accordingly, we propose the following hypothesis:

Hypothesis 3: Social capital mediates the relationship between physical exercise and subjective well-being, such that physical exercise indirectly enhances well-being by increasing social capital.

**Figure 1:**
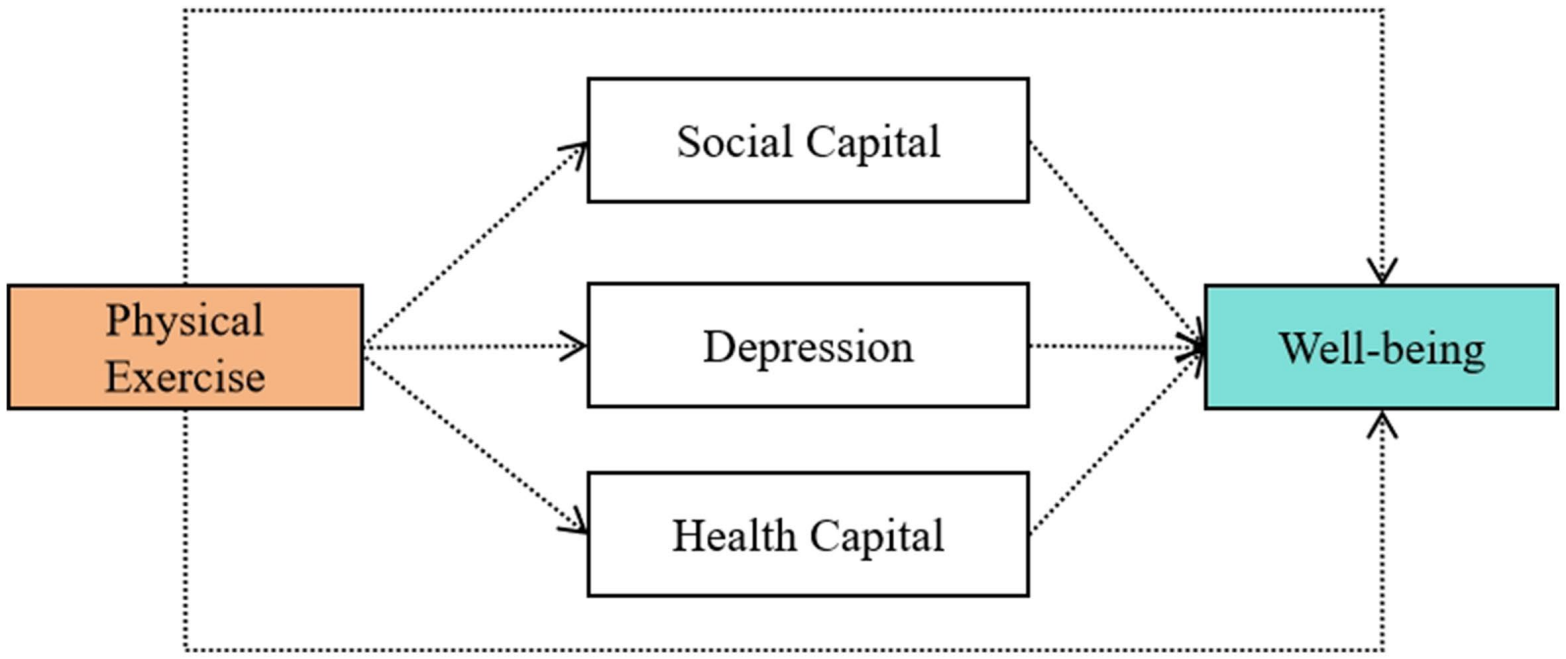
Conceptual model of the relationship between physical exercise and well-being.

Building upon the aforementioned research gaps and hypotheses, this study utilizes three waves of panel data from the China Family Panel Studies (CFPS) to systematically investigate the influence of physical exercise on subjective well-being. For the first time within a collectivist cultural context, we empirically compare whether the social pathway holds theoretical predominance and focus on assessing the relative strength of three mediating pathways: social capital, health capital, and depression risk. This aims to provide rigorous evidence on how cultural contexts shape the psychosocial mechanisms of health-related behaviors (Figure 1).

## 3 Data, variables, and methods

### 3.1 Data source

The data for this study are drawn from the China Family Panel Studies (CFPS), a nationally representative longitudinal survey administered by the Institute of Social Science Survey at Peking University. The CFPS comprehensively tracks socioeconomic, demographic, household, and health dynamics across China and is widely recognized for its credibility, national representativeness, and scientific rigor. It has been extensively utilized in research across disciplines such as sociology and psychology. To ensure analytical validity, we cleaned the data by removing observations with missing values on key variables and ambiguous responses. The final analytical sample comprises balanced panel data spanning three waves (2018, 2020, and 2022), covering 12,728 unique individuals and totaling 38,184 person-wave observations.

### 3.2 Variable selection

#### 3.2.1 Dependent variable: subjective well-being

The concept of Subjective Well-being (SWB), first introduced by Ed Diener in 1984, comprises both affective and cognitive dimensions. The affective component refers to the emotional experiences individuals encounter in daily life, including both positive and negative affect. The cognitive component pertains to individuals’ global judgment of their life satisfaction. Aligned with this conceptual framework and the design of the CFPS questionnaire, the present study measures well-being through a self-reported happiness item. The specific survey question is: “How happy do you feel?.” Responses are recorded on a Likert scale (1–10), with higher scores indicating stronger subjective well-being.

#### 3.2.2 Independent variable: participation in physical exercise

This study aims to examine the impact of regular physical exercise on subjective well-being. The China Family Panel Studies (CFPS) employed differing questionnaire items across survey waves: in 2018, respondents were asked “How many times did you exercise in the past week?” whereas in 2020 and 2022, the questions were, respectively, “How often did you participate in physical fitness or leisure activities in the past 12 months?” and “How many minutes did you usually spend on each exercise session in the past 12 months?” This inconsistency in measurement instruments poses a fundamental challenge for constructing a continuous variable that is precisely comparable across time.

Therefore, to generate an indicator with clear behavioral significance and maximal comparability, this study focuses on identifying individuals who have developed a habit of regular physical exercise. Regular exercise—typically defined as engaging in exercise multiple times per week for a sustained duration—is an important health behavior concept that has been widely demonstrated to correlate independently with positive physical and mental health outcomes (Bull et al., 2020; Powell et al., 2018). Drawing upon the common practice in existing research using CFPS data to define physical exercise (Xia et al., 2024; Wang et al., 2022), we operationalize physical exercise as a binary variable as follows: individuals who engage in physical exercise on average at least three times per week and exercise sessions last at least 30 min each are classified as having a “habit of regular physical exercise” and coded as 1; all others are coded as 0.

It should be noted that this represents a pragmatic and compromise approach constrained by the particularities of the available data. While it does not distinguish exercise intensity, loses some detail regarding exercise behavior, and is subject to potential recall bias, the core value of this study lies in testing the association between the “habit of regular exercise” and subjective well-being. In the face of measurement heterogeneity, this approach provides a methodologically feasible classification rule supported by precedent, effectively enhancing comparability across waves. To assess the potential impact of these limitations on the study findings, rigorous sensitivity analyses are conducted subsequently.

#### 3.2.3 Control variables

Following established literature and the questionnaire design, this study incorporates a set of control variables, including respondent’s gender, age, educational attainment, household registration (hukou) type, marital status, employment status, personal income, sleep duration, and out-of-pocket medical expenses. The specific coding schemes are as follows:

Gender: Male = 1, Female = 0. Age: A continuous variable, calculated as the survey year minus the respondent’s year of birth. Educational Attainment: Represented by the highest level of education completed, coded ordinally: Illiterate/Semi-illiterate = 1, Primary School = 2, Junior High School = 3, High School/Vocational School = 4, Associate Degree = 5, Bachelor’s Degree = 6, Master’s Degree = 7, Doctoral Degree = 8. Higher values indicate higher educational levels. Hukou Type: Urban = 1, Rural = 0. Marital Status: Unmarried/Cohabiting = 1, Married = 2, Divorced/Widowed = 3. Employment Status: Derived from the CFPS question: “Did you work for at least 1 hour in the past week?.” Yes = 1, No = 0. Personal Income: A continuous variable indicating the monthly after-tax income from the respondent’s primary job. Sleep Duration: A continuous variable. Out-of-pocket Medical Expenses: A continuous variable based on the question: “Excluding reimbursed or expected-to-be-reimbursed costs, how much did your household pay directly for medical expenses in the past 12 months?”

#### 3.2.4 Mediating variables

The mediating variables in this study include health capital, depression risk, and social capital.

Health capital: This variable is assessed using the survey item: “How would you describe your health status?.” Responses are coded on a 5-point scale: 1 = “Unhealthy,” 2 = “Fair,” 3 = “Relatively healthy,” 4 = “Very healthy,” 5 = “Extremely healthy.” Higher scores indicate better perceived health.

Depression risk: This variable is measured using the 8-item CFPS Depression Scale. Respondents indicate the frequency of specific feelings or behaviors over the past week, such as: “I felt depressed,” “I felt that everything I did was an effort,” “My sleep was restless,” “I was happy,” “I felt lonely,” “I enjoyed life,” “I felt sad,” and “I could not get going.” Response options are: “Rarely or none of the time (less than 1 day)” = 0, “Some or a little of the time (1–2 days)” = 1, “Occasionally or a moderate amount of the time (3–4 days)” = 2, “Most or all of the time (5–7 days)” = 3. Items worded positively are reverse-scored. The total score is calculated by summing the responses, with higher scores indicating a greater risk of depression.

Social capital: This construct is conceptualized as comprising three dimensions: social interaction, social status, and social trust. Social Interaction is measured by the item: “How would you rate your interpersonal relationships?.” Higher scores indicate better social connections. Social Status is assessed by the question: “How would you score your social status in your local area?.” Higher values reflect a higher self-perceived social standing. Social Trust is derived from the average level of trust respondents report towards six targets: parents, neighbors, Americans, strangers, the government, and doctors. The overall social capital score is calculated as the sum of the scores from these three dimensions.

### 3.3 Methods

This study employs the Bootstrap method based on Structural Equation Modeling (SEM) to test mediation effects (Preacher and Hayes, 2008). This approach constructs the empirical distribution of the indirect effect through repeated resampling and calculates 95% bias-corrected confidence intervals, offering advantages such as not relying on the assumption of normality and achieving higher statistical power (Hayes, 2017). First, we tested the mediating effects of depressive symptoms, health status, and social interaction separately using simple mediation models. Then, a parallel multiple mediation model was established to simultaneously examine the three mediation paths, controlling for the correlations among the mediators and comparing their relative importance (see Equations 1–4).


$$M_{1}=\alpha_{10}+\alpha_{11}X+\sum_{\kappa =1}^{p}\gamma_{1\kappa }C_{\kappa }+\varepsilon_{M_{1}}$$ (1)

$$M_{2}=\alpha_{20}+\alpha_{21}X+\sum_{\kappa =1}^{p}\gamma_{2\kappa }C_{\kappa }+\varepsilon_{M_{2}}$$ (2)

$$M_{3}=\alpha_{30}+\alpha_{31}X+\sum_{\kappa =1}^{p}\gamma_{3\kappa }C_{\kappa }+\varepsilon_{M_{3}}$$ (3)

$$Y=\beta_{0}+\beta_{1}X+\beta_{2}M_{1}+\beta_{3}M_{2}+\beta_{4}M_{3}+\sum_{\kappa =1}^{p}\gamma_{\kappa }C_{\kappa }+\varepsilon_{Y}$$ (4)

In these equations, *X* denotes physical exercise, *Y* denotes subjective well-being, M1, M2, M3 represent depression risk, health capital, and social capital, respectively, C_k_ is a vector of control variables, and ϵ denotes error terms.

## 4 Results

### 4.1 Descriptive statistics

Analysis based on the three-wave panel data (2018–2022) from the CFPS is presented in Table 1. The mean score of residents’ well-being is 7.49, a finding consistent with the theoretical framework of subjective well-being proposed by Diener et al. (2018). This suggests that the overall well-being of the Chinese population falls within the medium-to-high range, while also indicating substantial individual variation. Regarding physical exercise participation, only 27.2% of residents met the criterion of exercising at least three times per week for a minimum of 30 min per session. This reflects disparities among Chinese residents in terms of exercise awareness, access to sports facilities, and the allocation of leisure time. Demographic characteristics show that the average age of the sample is 46.007 years, with a mean education level of 3.048 (equivalent to junior high school). Rural household registration accounts for 72.5% of the sample, and the gender distribution is relatively balanced. Notably, the distribution of out-of-pocket medical expenses exhibits significant right-skewness (standard deviation substantially exceeds the mean). This pattern aligns with findings from Chu et al. (2018) regarding health shocks, revealing a pronounced inequality in the burden of healthcare costs among Chinese residents, which may have important implications for well-being.

**Table 1:** Descriptive statistics of variables.

Variable type	Variable name	Definition	Symbol	Std. Dev.	Mean
Dependent variable	Well-being	Self-rated happiness, higher values indicate greater happiness	Happiness	2.075	7.493
Independent variable	Physical exercise	=1 if exercise ≥3 times/week AND ≥30 min/session; =0 otherwise	Exercise	0.445	0.272
Control variables	Gender	Male = 1, Female = 0	Gender	0.5	0.496
	Age	Continuous variable (years)	Age	16.715	46.007
	Education	Illiterate/Semi-illiterate = 1, Primary School = 2, Junior High = 3, High School/Vocational = 4, Associate Degree = 5, Bachelor’s = 6, Master’s = 7, Doctorate = 8	Education	1.436	3.048
	Hukou type	Urban = 1, Rural = 0	Hukou	0.447	0.275
	Marital status	Unmarried/Cohabiting = 1, Married = 2, Divorced/Widowed = 3	Marriage	0.464	1.912
	Employment status	=1 if worked at least 1 h in the past week; =0 otherwise	Work	0.474	0.659
	Out-of-pocket medical expenses	Continuous variable (Yuan)	Medical_exp	8483.001	1951.595
Mediating variables	Health capital	1 = Unhealthy to 5 = Very healthy, higher values indicate better health	Health	1.199	3.074
	Depression risk	CES-D 8-item scale, higher scores indicate greater risk	Depression	4.2	13.507
	Social capital	Composite index (sum of scores for relationship, trust, and position)	Soc_capital	3.096	15.655
	Social interaction	Self-rated interpersonal relationships, higher scores indicate better relationships	Relationship		
	Social trust	Average trust in parents, neighbors, Americans, strangers, government, doctors	Trust		
	Social status	Self-rated social status in local community, higher scores indicate higher status	Position		

### 4.2 Analysis of the direct effect of physical exercise on well-being

The results from the pooled regression models, presented in Table 2, provide strong support for Hypothesis 1. Model 1 shows that the crude effect of physical exercise on well-being is 0.369 (*p* < 0.001). This indicates that, without controlling for any other factors such as age, gender, or education level, individuals who participated in physical exercise reported an average well-being score 0.369 points higher than those who did not. This finding aligns closely with the physiological mechanism proposed by Basso and Suzuki (2017), whereby exercise influences mood through the regulation of neurotransmitters. In terms of effect size, participation in physical exercise increases individual well-being by approximately 0.37 standard deviations, an effect considered to be of substantial practical significance.

**Table 2:** Results from pooled regression models analyzing factors influencing well-being.

Variable	Model 1	Model 2	Model 3
Exercise	0.369^***^	0.307^***^	0.095^***^
Age		0.007^***^	−0.001
Gender		−0.021	−0.109^***^
Education		0.030^***^	−0.031*
Hukou		0.096^***^	0.149^***^
Marriage		−0.209^***^	0.012
Work		−0.136^***^	−0.159^***^
Medical_exp		−0.000^***^	0.000^***^
Depression			−0.116^***^
Health			0.145^***^
Soc_capital			0.291^***^
_cons	7.393^***^	7.493^***^	4.256* ^***^
*F*	275.850	61.781	1804.138

^***^p < 0.01, ^**^p < 0.05, ^*^p < 0.1.

After controlling for demographic variables (Model 2), the coefficient for physical exercise attenuates to 0.307 (*p* < 0.001), representing a reduction of 16.8%. This attenuation pattern suggests two plausible interpretations. On one hand, demographic characteristics may partially mediate the relationship between physical exercise and well-being. For instance, higher educational attainment likely promotes both participation in physical exercise (due to greater health awareness and resources) and directly enhances well-being, a pattern consistent with prior research indicating higher exercise participation rates among highly educated populations (Guthold et al., 2018). On the other hand, the attenuation also hints at the potential presence of selection bias. That is, the probability of participating in physical exercise is not random across individuals; those with more advantageous socioeconomic backgrounds (e.g., urban residence, higher income) may possess greater capacity and motivation for regular exercise, and these background factors are themselves associated with higher well-being. Consequently, the crude effect observed in Model 1 may overestimate the impact of physical exercise. By incorporating control variables, Model 2 partially isolates these confounding factors, presenting a more refined estimate of the explanatory pathway through which physical exercise influences well-being. This finding prompts further consideration: beyond these relatively stable demographic characteristics, what other psychological and social factors might be at play?

Of particular note, after introducing the three mediating variables (Model 3), the coefficient for physical exercise further decreases to 0.095 (*p* < 0.001), accounting for only 25.7% of the original crude effect. This substantial attenuation provides preliminary evidence for the existence of multiple mediating mechanisms. On one hand, it indicates that a significant portion of the impact of physical exercise on well-being is transmitted through the mediating variables incorporated in the model. On the other hand, the direct effects of all three mediators on well-being are highly significant. This implies that improvements in health, social interaction quality, and psychological state—triggered by participation in physical exercise—can indeed serve as effective pathways for enhancing well-being. These findings support the theoretical framework proposed by Mcauley et al. (2005), which posits that physical exercise influences well-being through multiple psychological mechanisms, such as self-efficacy. In essence, the benefits of physical exercise are not conveyed through a single physiological pathway but are realized through a combination of multiple psychological and social mechanisms.

### 4.3 Robustness checks using panel data models

Panel data models provide more rigorous evidence regarding the relationship between physical exercise and well-being by controlling for unobserved factors across different dimensions. First, the time-fixed effects model (Model 4) yields a coefficient for physical exercise of 0.300 (*p* < 0.001), which is similar to the pooled regression results. This suggests that time trends do not confound the primary relationship under investigation.

The results of the individual fixed-effects model (Model 5) show that after controlling for time-invariant individual characteristics, the coefficient for physical exercise sharply attenuates to 0.035 and becomes statistically insignificant. This finding poses a serious challenge to the causal inference in this study. The individual fixed-effects model is widely regarded as a more rigorous approach for identifying causal effects in observational data. The results of Model 5 suggest that the significant association between physical exercise and subjective well-being observed in simpler models may largely reflect stable differences between individuals. For example, individuals who are inherently more positive in affect and more self-disciplined may simultaneously exhibit higher baseline well-being and more consistent exercise habits (Moor et al., 2006). This does not imply that the association between physical exercise and subjective well-being lacks research value, but rather calls for a more nuanced understanding of the nature of this association: specifically, there exists a “robust statistical association” between physical exercise and well-being, and elucidating the pathways through which this association arises is of equal theoretical importance. If the association is primarily driven by stable traits, through which observable psychosocial processes do these traits influence well-being? If there is a modest within-individual causal effect, what mechanisms mediate this effect? These questions constitute the core issues addressed by the subsequent mediation analysis. By testing potential pathways such as social capital, health capital, and depression risk, the following sections will explore in depth the process by which the association is formed. This approach provides richer insights into the sources of variation in subjective well-being.

The two-way fixed effects model (Model 6), which controls for both individual and time fixed effects, provides the most stringent causal evidence. This model captures the effect of changes within the same individual over time. The coefficient for physical exercise is 0.048, significant at the 10% level. This result confirms that when an individual increases their level of physical exercise, their well-being does indeed improve, although the magnitude of this effect is substantially smaller than that suggested by cross-sectional comparisons. This finding offers the most robust, albeit cautious, support for Hypothesis 1.

Furthermore, the changes in the coefficients of the control variables across models underscore the significant impact of model specification on the interpretation of results. In Model 4 (time-fixed effects), educational attainment and hukou type exhibit statistically significant positive effects on well-being. However, in Models 5 and 6, which control for individual fixed effects, the coefficients for these time-invariant variables are absorbed by the fixed effects or become statistically insignificant. In contrast, the negative association between marital status and well-being remains robust across different model specifications. These comparisons highlight that failing to account for the multi-level structure of panel data can lead to a misinterpretation of variable effects. Specifically, cross-sectional associations may conflate the roles of stable individual characteristics with the dynamic effects of time-varying factor (see Table 3).

**Table 3:** Results from panel data model estimations.

Variable	Model 4: Time-fixed effects	Model 5: Individual-fixed effects	Model 6: Two-way fixed effects
Exercise	0.300^***^	0.035	0.048*
Age	0.007^***^	−0.029^***^	0.019
Gender	−0.021	0.328	0.321
Education	0.032^***^	−0.011	−0.014
Hukou	0.095^***^	−0.029	−0.033
Marriage	−0.212^***^	−0.169^***^	−0.167^***^
Work	−0.137^***^	−0.001	0.000
Medical_exp	−0.000^***^	−0.000^***^	−0.000^***^
2018.year	0.000		0.000
2020.year	0.021		−0.047
2022.year	−0.092^***^		−0.193
_cons	7.509^***^	9.013^***^	6.886^***^
*F*	51.851	9.230	8.521

^***^p < 0.01, ^**^p < 0.05, ^*^p < 0.1.

### 4.4 Empirical analysis of the mediating mechanisms

The Bootstrap mediation effect analysis reveals a total effect of physical exercise on well-being of 0.314 (*p* < 0.001) (see Table 4). Building on this, we further decompose this total effect into the direct effect and the indirect effects transmitted through different mediating variables. This decomposition provides differentiated empirical support for Hypotheses 2, and 3.

**Table 4:** Bootstrap mediation effect analysis results.

Variable	Total effect	Total effect S.E.	Direct effect	Direct effect S.E.	Indirect effect	Indirect effect S.E.	95% CI lower bound (Indirect effect)	95% CI upper bound (Indirect effect)	Significant (95% CI)
Depression	0.314^***^	0.023	0.191^***^	0.021	0.122^***^	0.008	0.105	0.150	Yes
Health capital	0.314^***^	0.023	0.270^***^	0.022	0.044^***^	0.005	0.033	0.066	Yes
Social capital	0.314^***^	0.023	0.172^***^	0.020	0.130^***^	0.011	0.119	0.135	Yes

^***^p < 0.01, ^**^p < 0.05, ^*^p < 0.1; Total effect = Direct effect + Indirect effect; If the 95% confidence interval does not include zero, the effect is considered statistically significant.

The indirect effect through the health capital pathway is 0.044 (*p* < 0.001), accounting for 14.0% of the total effect. This finding offers partial support for Hypothesis 2, confirming that health capital serves as a significant pathway through which physical exercise influences well-being—a conclusion consistent with the findings of Wang et al. based on CGSS data (Wang et al., 2021). It is noteworthy, however, that although the mediating effect of health capital is statistically significant, its relative magnitude is modest. This observation aligns with the results from the individual fixed effects model (Model 5) discussed earlier, where the well-being effect of physical exercise substantially attenuated after controlling for time-invariant individual heterogeneity. This pattern suggests that improvements in purely physiological health may not constitute the core mechanism through which physical exercise enhances well-being.

The social capital pathway exhibited the strongest mediating effect, with an indirect effect of 0.140 (*p* < 0.001), accounting for 44.6% of the total effect—3.2 times that of the health capital pathway. This finding supports Hypothesis 3 and reveals a distinctive mechanism in the Chinese socio-cultural context whereby the influence of physical exercise on subjective well-being manifests a “social precedence over physiological” characteristic. Possible explanations include: First, modulation by cultural values. Chinese culture is characterized by collectivism and high-context communication (Hofstede, 2011), where well-being is deeply embedded in social relationships and social evaluation (Lu and Gilmour, 2004). Against this backdrop, the social interactions, group belonging, and relational harmony derived from physical exercise directly fulfill core psychological needs for “relationship integration” and “social identity.” In collectivist cultures, social support and social integration are significantly stronger predictors of subjective well-being than personal achievement and autonomy. Hence, the social attributes of physical exercise more effectively tap into the core sources of well-being in Chinese culture than mere physiological health improvements. Second, structural advantages of social capital. According to social capital theory (Putnam, 2000), Chinese society particularly depends on “strong-tie” networks based on family, neighborhood, and hometown connections, which give rise to informal support systems. Physical exercise—particularly collective activities conducted in public spaces such as parks, community squares, and workplace sports facilities (e.g., square dancing, Tai Chi, brisk walking)—naturally serves as a platform for constructing and strengthening such social capital. These activities foster interpersonal trust and norms of reciprocity, providing valuable social integration and emotional support amid rapid social change, thereby significantly buffering stress and enhancing well-being. By contrast, health capital (self-rated health) improvements, though important, primarily affect individuals at a personal level and may be overshadowed by the stronger interpersonal and collective mobilization capacities of social capital within China’s “relationship-oriented” societal structure. Third, sense of belonging and meaning from collective exercise. In China, many forms of physical exercise have been internalized as “collective rituals.” For example, square dancing is not only a form of fitness but also a crucial social occasion for middle-aged and elderly women to rebuild social connections and gain community recognition. These activities adhere to cultural scripts of “maintaining harmonious relationships through collective activities,” with social functions arguably taking precedence over fitness purposes. Thus, participating in physical exercise itself can be regarded as conforming to social norms and reinforcing social integration, directly generating a sense of belonging and meaning (Steptoe and Fancourt, 2019). Such psychological benefits may yield more immediate and potent effects on well-being than physiological improvements.

Depression risk also showed a substantive mediating effect, with an indirect effect value of 0.122, accounting for 38.9% of the total effect, second only to social capital. This result supports Hypothesis 2. Steptoe et al. (2015) confirmed that depressive mood is one of the most stable and powerful negative predictors of subjective well-being. Any factor effectively reducing depression risk naturally becomes a vital pathway for enhancing well-being, and the proportion of the mediation effect (38.9%) quantifies the strength of this relationship. Chekroud et al. (2018), based on a study of 1.2 million individuals, also found a significant dose–response relationship between physical exercise and mental health (including depressive symptoms), with exercise benefits on mental health independent of demographic and socioeconomic factors. Our findings align with this large-sample evidence, further supporting the pathway from physical exercise → reduced depression risk → increased subjective well-being. It is noteworthy that in Model 5 (individual fixed effects), the coefficient of exercise becomes non-significant (0.035). This finding suggests that the impact of physical exercise on well-being may partly operate through relatively stable within-individual traits or long-term tendencies. As a mediator, depression risk itself exhibits high individual stability (e.g., genetic predisposition, personality bases, or chronic psychological traits). Therefore, a plausible explanation is that controlling for individual fixed effects (i.e., removing all time-invariant individual differences) partially eliminates the “long-term benefits” of physical exercise related to these stable traits, thereby attenuating its short-term “net effect.” This result is consistent with theoretical expectations that depression risk mediates the long-term psychological benefits of exercise as a stable trait, but it also implies the possible existence of other time-invariant confounders (such as genetic or early-life environmental factors) that jointly influence physical exercise, depression, and well-being. Future research should employ more rigorous longitudinal designs or genetic data analyses to clarify these causal pathways.

## 5 Discussion

### 5.1 In-depth explanation of the central mediating role of social capital

The core contribution of this study lies in establishing, with quantitative evidence within the Chinese socio-cultural context, the dominant mediating role of social capital in the relationship between physical exercise and well-being. This finding is not incidental but deeply rooted in China’s collectivist culture, relationship-oriented social structure, and the ongoing social transition.

First, from the perspective of cultural-psychological foundations, collectivist culture shapes a self-concept grounded in “relationality” and “interdependence,” where an individual’s well-being is closely linked to the harmony of social relationships and the fulfillment of group belonging. In the Chinese cultural setting, physical exercise is often not merely an individual activity but a socially embedded practice. Whether it is community square dancing, Tai Chi groups, or various organized team competitions, these activities carry profound social significance—they serve as important venues for building and maintaining relationships, fulfilling social roles, and gaining group recognition (Liang and Jia, 2022). Therefore, the social connections and trust reinforced through participation in collective physical activities directly satisfy core psychological needs for “belonging” and “relational harmony” among Chinese people, naturally producing stronger well-being effects. This explains why the mediating effect of social capital far exceeds that of the “health capital” pathway, which focuses more on individual physiological status.

Second, from the perspective of social structural foundations, within China’s “differential mode of association” social structure, social capital is a key resource for obtaining support, information, and opportunities. Communities play a vital role in social integration and support in both urban and rural Chinese life. Community-based physical exercise activities—such as neighborhood fitness teams and park activity spots—have become valuable platforms in contemporary society for activating and consolidating this localized social capital (Huang et al., 2024). Through regular participation, individuals not only exercise their bodies but also weave and maintain a micro social support network grounded in geographic proximity. This network functions in everyday mutual aid, emotional support, and even crisis response, thereby significantly enhancing subjective well-being. In contrast, in more individualistic societies, individuals tend to depend less on community, and the well-being effects of physical exercise may primarily derive from improvements in personal physical functioning.

Finally, viewed against the backdrop of social transition, China is undergoing rapid urbanization and societal transformation, with traditional social bonds loosening and digital technologies potentially exacerbating social isolation. In this context, offline physical exercise activities have their social integrative value unprecedentedly amplified due to their ability to provide scarce, high-quality face-to-face social opportunities (Holt-Lunstad, 2022). Participation in physical exercise has become an active choice for many urban residents to combat loneliness and rebuild social connections. Thus, the dominance of the social capital pathway can also be seen as a proactive compensation for social alienation amid social change, rendering the “social healing” function of physical exercise especially prominent and effective in contemporary China.

### 5.2 Theoretical contributions

#### 5.2.1 Theoretical advancement: from individual behavior to social relationship practice

This study quantitatively establishes the dominant mediating role of social capital in the relationship between physical exercise and well-being, thereby echoing and extending theories of social integration and relational perspectives. Traditional health behavior theories tend to overemphasize individual cognition, attitudes, intentions, and decision-making, often neglecting the social structures and cultural contexts in which health behaviors occur (Hagger and Hamilton, 2025). Our findings demonstrate that in a highly collectivist and relationship-oriented society like China, the core mechanism through which physical exercise enhances well-being lies in its function as a “catalyst for social relationships” — it creates shared time and space, strengthens community bonds, and fosters trust and reciprocity, fulfilling fundamental human needs for belonging and social identity (Liang and Jia, 2022). This insight aligns with the recently emerging “Relational Health” theory (Holt-Lunstad, 2022), which highlights the central role of social connections in health promotion and constitutes a significant revision to traditional individualistic health models. Therefore, any health behavior theory that neglects the social context risks incompleteness when explaining well-being phenomena in collectivist cultures. Our findings further advance the field’s paradigmatic shift from a conventional “individual health behavior” perspective toward a more integrative “social relationship practice” framework.

#### 5.2.2 Methodological implications: cautionary lessons on causal inference in observational studies

The differing results between the fixed effects and mixed models offer valuable methodological warnings regarding causal inference in observational research. Although fixed effects models are advantageous in controlling for time-invariant heterogeneity, the attenuation of coefficients suggests that the observed cross-sectional associations are likely confounded by stable individual traits that simultaneously influence both physical exercise and perceived well-being—such as extraversion, genetic predispositions, or childhood experiences (Kathryn and Rodney, 2015). This compels us to consider more complex causal explanatory frameworks. The well-being benefits of physical exercise may result from two parallel mechanisms: long-term shaping of positive personality traits or lifestyle, and “short-term causal” effects through immediate social interactions. This finding resonates with recent discussions in causal inference methodology emphasizing that causal claims in observational studies require theoretically informed designs combined with multiple modeling approaches (Fu, 2023).

### 5.3 Practical implications

The findings of this study not only reshape our theoretical understanding of health behavior mechanisms but also provide precise practical guidance for designing effective health promotion strategies within the Chinese socio-cultural context. On one hand, given the dominant role of social capital, public health initiatives should shift from merely “encouraging individual exercise” to “building exercise-friendly communities” (Ren et al., 2025). Policy designs ought to emphasize the dual embedding of physical activity and social connections, such as promoting community sports festivals, neighborhood exercise groups, and intergenerational physical activity programs, to strengthen social capital’s role. On the other hand, differentiated exercise intervention policies should be tailored to various populations, especially targeting high-risk groups—such as the elderly, low-income individuals, and socially isolated persons—with integrated interventions combining social support and physical activity (Liang and Jia, 2022). Furthermore, the significance of the health capital pathway supports the integration of physical exercise into primary healthcare systems to fully harness its potential in chronic disease prevention and health management.

## 6 Conclusion

### 6.1 Main findings

This study utilizes panel data from three waves of the China Family Panel Studies (CFPS) spanning 2018 to 2022, and employs a combination of mixed regression models, fixed effects models, and Bootstrap mediation tests to systematically examine the impact of physical exercise on well-being and its underlying mechanisms. The findings are as follows: First, there is a robust association between physical exercise and well-being; however, results from the fixed effects model suggest that this relationship may partly stem from stable individual traits, posing challenges to straightforward causal inference. Second, social capital serves as the strongest mediating pathway through which physical exercise influences well-being, with its mediating effect significantly surpassing those of health capital and depression risk pathways.

### 6.2 Limitations and future directions

This study has several limitations. First, although panel data were employed to provide more robust evidence on the association between physical exercise and subjective well-being than cross-sectional studies, causal inference remains limited. Specifically: (1) Reverse causality cannot be fully ruled out. While theoretical models and social-cognitive research predominantly support the pathway “physical exercise promotes well-being,” the competing explanation that individuals with higher well-being are more motivated or able to participate in physical exercise cannot be completely excluded. (2) The models cannot control for unobserved time-varying factors that simultaneously influence physical exercise and well-being—such as household economic shocks, health events, or changes in neighborhood social capital. (3) Core variables, including physical exercise and social capital, despite being based on validated scales or conventional measures, are still subject to measurement error. Hence, this study primarily reveals a robust statistical association between physical exercise and well-being, and causal interpretations should be made cautiously. Second, limitations exist in the measurement of physical exercise. Although we adopted a binary definition of “regular participation in physical exercise” based on theoretical and methodological precedents to address inconsistencies in measurement tools across survey waves, this definition cannot precisely capture the total volume or intensity of activity, and recall bias may be present. While multiple sensitivity analyses have supported the robustness of the findings, future research employing standardized instruments combined with objective measurements will enable more precise examination of different dimensions of physical exercise (frequency, duration, intensity) in relation to well-being. Third, measurement tools may present limitations as well, particularly regarding the sensitivity of the CES-D scale in community samples for capturing depression risk (Fried, 2017).

Future research could advance this field in several ways. First, to address reverse causality and time-varying confounding, exogenous instrumental variables or quasi-experimental designs using policy shocks (e.g., difference-in-differences methods) may be applied to identify the local average treatment effect of physical exercise. Whenever feasible, randomized controlled trials remain the gold standard for evaluating causal effects. Second, regarding mechanism testing, longitudinal data with multiple waves should be utilized alongside advanced methods such as random intercept cross-lagged panel models to rigorously assess the temporality and directionality of mediation pathways. Third, conducting in-depth heterogeneity analyses to explore differences in main effects and mediation pathways across diverse sociocultural contexts will provide critical evidence for designing targeted public health interventions.

## Data Availability

Publicly available datasets were analyzed in this study. This data can be found here: http://www.isss.pku.edu.cn/cfps/index.htm.
